# A Unique Case of Eosinophilic Pancreatitis and Anencephaly in the Fetus of a Type I Diabetic Mother

**DOI:** 10.4021/gr332w

**Published:** 2011-07-20

**Authors:** Elias R. George, Shirali S. Patel, Priyanka Sen, Norbert Sule

**Affiliations:** a Department of Pathology and Immunology, Baylor College of Medicine, One Baylor Plaza, M315, Houston, TX 77030, USA; b Department of Student Affairs, Baylor College of Medicine, One Baylor Plaza, BCM368, Houston, TX 77030, USA; c Department of Department of Pathology, Roswell Park Cancer Institute, Elm and Carlton Streets, Buffalo, NY 14263, USA; d Conresponding author: Norbert Sule, Roswell Park Cancer Institute, Buffalo, NY 14263, USA. Email: norbert.sule@roswellpark.org

**Keywords:** Eosinophilic pancreatitis, Anencephaly, Type 1 diabetes, Diabetic mother, Neural tube defect, Fetus, Stillborn

## Abstract

Pancreatic infiltration with eosinophils is an uncommon finding with numerous etiologies. While two rare cases of eosinophilic pancreatitis in infants born to Type I diabetic mothers have been reported once in the English literature and once in the French literature, we present the additional finding of anencephaly in a 34 week old fetus. Although the pancreas was grossly unremarkable, histological inspection demonstrated an eosinophilic infiltrate in the fibrous septae and islets of Langerhans along with hypertrophy and hyperplasia of the pancreatic islets.

## Introduction

Pancreatic infiltration with eosinophils is an uncommon finding with many etiologies including lymphoplasmacytic sclerosing pancreatitis (LPSP), pancreatic allograft rejection, pancreatic pseudocyst, inflammatory myofibroblastic tumor, histiocytosis X and most rarely, eosinophilic pancreatitis [1]. In adults and adolescents, eosinophilic pancreatitis often presents with obstructive symptoms suggestive of a pancreatic neoplasm [2]. Commonly, the diagnosis is not made until pancreatic resection is performed [2], and has been reported more in adults, in whom malignancy, parasitic infection, hypersensitivity response to medications (carbamazepine, toxin injection), and milk allergy have been found as causative agents [1]. Of note, rare cases of eosinophilic pancreatitis demonstrating eosinophilic infiltration associated with islet hypertrophy have been reported in infants born to Type I diabetic mothers [3].

Neural tube defects have been reported to occur worldwide in approximately 1 to 10 per 1,000 births [4]. Interestingly, anencephaly and spina bifida occur more commonly in infants born to women with diabetes [5, 6]. Here we present a unique case of a stillborn fetus delivered by a Type I diabetic mother with both eosinophilic pancreatitis and anencephaly.

## Case Report

After confirming intrauterine fetal demise by ultrasound at 34 4/7 weeks gestation, a female fetus was delivered vaginally by a 21 year old G4P2 Hispanic woman with no access to prenatal care. The mother had a history of juvenile onset diabetes mellitus, which was reportedly being managed with metformin and insulin. HgbA1c measured one month prior to delivery, however, was 8.8%. She denied taking any other medications during pregnancy. At delivery, her serologies for rubella, hepatitis B, *Neisseria gonorrhoea* and *Chlamydia trachomatis* were negative, as was her urine drug screen. Also, she denied any history of asthma, allergies, or vasculitides.

**Figure 1:**
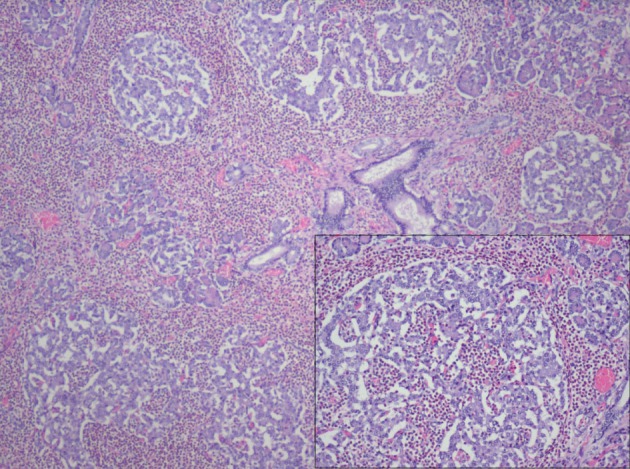
40X magnification, H&E: Hypertrophy and hyperplasia of the pancreatic islets of Langerhans with prominent septal, peri-insular and islet eosinophilic infiltrate (see insert, 100X magnification) limited to pancreatic parenchyma with relative sparing of the exocrine pancreas.

After consent was obtained, autopsy of the stillborn fetus was performed. Major findings on gross inspection include anencephaly and mild left renal hypoplasia. No other gross abnormalities were identified. Of particular note, the pancreas was in its normal anatomical position with no identifiable lesions. On routine histology, hypertrophy and hyperplasia of the pancreatic islets of Langerhans with prominent septal, peri-insular and islet eosinophilic infiltrate with relative sparing of the exocrine pancreas was noted (Fig. 1). The eosinophilic infiltrate was limited to the pancreatic parenchyma and not noted in histological sections of the other organs. No arteritis or phlebitis was present. Examination of the placenta and umbilical cord revealed three vessels with funisitis and marked acute inflammation was present indicative of chorioamnionitis. However, no features suggestive of chromosomal pathology or gestational trophoblastic disease were seen.

## Discussion

In the English literature, Barresi et al have previously described the case of an infant born at 34 weeks gestation to a diabetic mother who had been on insulin therapy for three years. An additional case report by Payan, et al from the French literature also describes a case of eosinophilic cell pancreatitis in newborn infants of diabetic mothers [7]. To the best of our knowledge, no previous case report has documented the extremely rare concurrence of anencephaly and eosinophilic pancreatitis in a fetus.

In the Barresi case report, the infant developed spontaneous tremors of the legs soon after birth and died from progressive respiratory distress resulting from bilateral pulmonary atelectasis with complicating hyaline membrane disease [3]. As in this case, the infant from the previous case report had a structurally normal pancreas with peri-insular eosinophilic leukocyte infiltration and hypertrophy and hyperplasia of islets of Langerhans [3]. Abraham et al reported two histologic patterns associated with eosinophilic pancreatitis in their three patients. One pattern was described as “localized eosinophilic infiltrates associated with pseudocyst formation”, and the second pattern was a “diffuse periductal, acinar, and septal eosinophilic infiltrate with eosinophilic phlebitis and arteritis” [1]. The pattern seen in our patient, while more similar to the latter description, has two striking differences. The eosinophilic infiltrate was not seen in the vasculature or acini; however, it was prominent in the fibrous septae and islets of Langerhans, suggesting a more focused distribution.

One possible mechanism of islet cell hyperplasia with eosinophilia was described by Barresi et al and involves the formation of materal IgG autoantibodies against insulin. These immunoglobulins then cross the placenta and couple with fetal insulin. It is these antigen-antibody complexes that then stimulate eosinophilia, whereby the eosinophils are believed to be involved in their phagocytosis [3].

Previous cases of pancreatic eosinophilic infiltration have been linked to peripheral eosinophilia in adults [1]. This association may hold true in fetal and pediatric cases as well. Given the limited involvement to the pancreas and sparing of the other organs and tissues, it is unlikely that peripheral eosinophilia existed in this case. Unfortunately, the peripheral eosinophil count was not obtained.

Neural tube defects have been reported to occur in higher incidence among Hispanic women, particularly those with lower education levels [5]. Although the educational background of our patient’s mother is unknown, her lack of prenatal care and her confirmed history of poorly controlled Type I DM suggest that her fetus was at increased risk for neural tube defects. Since no record of the mother’s folate levels during early pregnancy exist, folate deficiency as the sole cause or as a contributing cause of anencephaly cannot be ruled out. In this case, a clear etiology for a relation between the findings of eosinophilic pancreatitis and anencephaly remains elusive.
